# Professional competencies in geriatric nursing for geriatric nurses: a latent profile analysis

**DOI:** 10.1186/s12912-024-02157-8

**Published:** 2024-07-29

**Authors:** Mengxue Wang, Dongdong Li, Jingjing Li, Xiumei Zhang

**Affiliations:** 1 Nursing Department, Anhui Medical University, 81 Meishan Road, Hefei, Anhui 230000 China; 2 Department of Nursing, The First Affiliated Hospital of Anhui Medical University, 210 Jixi Road, Hefei, Anhui 230000 China; 3 Department of Nursing, The First Affiliated Hospital of USTC, Division of Life Sciences and Medicine, University of Science and Technology of China, Western District, 107 East Huanhu Road, Hefei, Anhui 230031 China

**Keywords:** Clinical nurses, Geriatric nursing, Competency, Latent profile analysis

## Abstract

**Background:**

As the global population continues to age, social realities such as advanced age, disability and living alone are coming to the fore, and the demand for medical care and health services for the elderly is increasing dramatically, especially in geriatrics. Given the important role geriatric nurses play in the diagnosis and treatment of diseases and rehabilitation of elderly patients, and due to the uniqueness and complexity of geriatric work, this requires geriatric nurses not only to have the competencies that are available in general nursing, but also to ensure that they have sufficient geriatric core competencies in order to effectively meet the needs of the patients and accelerate their recovery. Although previous studies have investigated the core competencies of nursing staff, there has been little research on geriatric nurses’ core geriatric nursing competencies and their predictors. The aim of this study was to investigate the current status of the geriatric nursing competency inventory (GNCI) among geriatric nurses using latent profiling, to identify potential subgroups and their population characteristics, and to explore the factors that influence the potential subgroups.

**Methods:**

From January to March 2024, 1,313 geriatric nurses in Hefei City were selected by stratified cluster sampling method and surveyed with general information questionnaire, geriatric nursing competency inventory, and occupational coping self-efficacy scale for nurses(OCSE-N). Potential subgroups of GNCI differences among geriatric nurses were identified by latent profile analysis (LPA). Multiple logistic regression analyses were used to explore the factors influencing the GNCI of geriatric nurses with different latent profiles.

**Results:**

Geriatric nurses’ OCSE-N was positively correlated with GNCI, and the GNCI score was 123.06(41.60), which indicated that geriatric nurses’ GNCI was at an intermediate level. The OCSE-N score was 35.44(7.34), which was at a relatively high level. There was heterogeneity in the GNCI of geriatric nurses, which was classified into three subgroups i.e., Low-competency group, Medium-competency group, High-competency group. The results of multiple logistic regression analyses showed that OCSE-N, title, whether or not they attended geriatric nurse specialist training, and specialist nurse status were predictors of GNCI among geriatric nurses (*P* < 0.05).

**Conclusion:**

The GNCI categorical characteristics of geriatric nurses are obvious, and nursing managers should adopt targeted interventions according to the characteristics of nurses in different profiles to improve the overall quality of care.

## Introduction

By the end of 2021, the global population aged 65 and over was 761 million, and this number is expected to increase to 1.6 billion in 2050, with the population aged 80 and over growing even faster [1]. Currently, China has the largest elderly population in the world, and this number is growing. It is expected that in 2050, China’s population aged 65 years and over will reach 395 million, which is equivalent to 1.2 times the current population of the United States. The oldest old (80^+^) will reach 135 million, more than the current population of Japan [1]. In addition, 81.3% of adults aged 60 years or older suffer from atleast one common chronic disease, and the co-morbidity rate continues to increase with age [2], and the average number of hospitalisations, length of stay, and economic costs are much higher than those of other groups, resulting in serious impairment of physical and mental function and quality of life for patients and their families, and further aggravating the burden on families, health care, and the social economy [1–3]. Thus, the elderly have become a major user group seeking health care, and how to effectively meet the growing demand for high-quality, multilevel health care services for the elderly has become a social and public problem that needs to be solved urgently.

Geriatric nurses, as one of the clinical workers with the longest contact time and most frequent interactions in the diagnosis and treatment of diseases and rehabilitation of elderly patients, are able to dynamically grasp the changes in the patients’ conditions, provide quality nursing services in a timely manner to meet the patients’ needs, and play an important role in ensuring the physical and mental health of the elderly patients [4]. The unique and complex nursing needs of the geriatric population cover medical, cognitive, emotional, social and environmental domains, and the signs and symptoms of their various diseases are significantly different from those of other age groups, which requires geriatric nurses not only to have the competencies that are available in general nursing, but also to ensure that they have adequate gerontological core competencies in gerontological nursing [5]. GNCI refers to the knowledge, skills and attitudes, as well as the legal and ethical practices, necessary for nurses to fulfil the range of roles of a professional nurse in the practice of geriatric nursing [6]. It is the basic competence required for nursing practice in the field and a key element in ensuring the quality of geriatric nursing care. In November 2021, China’s Health and Wellness Commission issued the notice on the pilot work of elderly medical care services, which clearly states that medical institutions should be instructed to provide multi-level geriatric medical care services for the elderly in accordance with their functional positioning and classified according to need, based on the characteristics of the illnesses, self-care ability and medical care needs of the elderly in the region [7]. Clinical nurses, as direct providers of nursing services, have a direct bearing on the efficiency and quality of services, as well as on the service experience and health outcomes of the elderly [8, 9]. In recent years, healthcare workers have also paid more attention to the GNCI levels of nurses and endeavoured to explore ways to improve them. Relevant training courses have been established and corresponding evaluation criteria have been developed [10–14]. These studies found that continued participation in training is a key initiative to improve the GNCI level of geriatric nurses, while experts pointed out that the improvement of advanced nursing practice skills and knowledge in geriatric nursing is the key to the enhancement of core competencies, and suggested that future training should focus on advanced geriatric nursing assessment and nursing diagnosis, counselling and guidance and geriatric patient education, geriatric disease and daily life management, management and organisation, and take skill operation training and clinical practice as the main forms to effectively improve the GNCI level of geriatric nurses [12]. In addition, existing studies have identified the importance of core competencies in geriatric nursing [15], tested the reliability and validity of self-administered core competency assessment scales for geriatric nurse specialists [16], and assessed the current status of core competencies for geriatric nurse specialists through qualitative methods [17]. However, geriatric nursing core competency measures tend to judge the level of core competency by the total scale score, ignoring inter-individual differences and possible group heterogeneity among the influencing factors due to different levels of core competency.

LPA is a probability-based classification of populations, which divides individuals into different categories based on their scores on various dimensions, and helps to explore the characteristics and influencing factors of different categories of populations [18]. With the same GNCI scores, there were significant differences in the importance attached to the GNCI by different nurses. Some nurses have stronger critical thinking and research skills or clinical nursing skills, while others have better leadership or interpersonal skills. Therefore, this study explored the differences in the distribution of geriatric nurses’ GNCI characteristics across dimensions using LPA and examined the effects of demographic variables on their different potential categories, which in turn explored the differences between categories among the variables to inform nursing managers in developing precise interventions to enhance nurses’ GNCI.

In addition, relatively few articles have been written on the factors influencing GNCI, focusing mainly on demographics, which are relatively homogenous. It has been suggested that self-efficacy affects the way individuals experience events in the workplace and their stress responses, and that individuals with high self-efficacy are able to effectively cope with work stress, burn out, and adverse emotions, handle interpersonal relationships flexibly, and develop positive work attitudes and commitment, which motivates them to put more effort into their work, thus affecting core competencies at work [19, 20]. The OCSE-N is a measure of self-efficacy related to the nursing worker-specific self-efficacy, i.e., the subjective evaluation and perception of nursing workers’ ability to effectively cope with and competently perform nursing tasks, which can directly influence nurses’ work attitudes and behaviours, thus affecting the overall quality of care [21]. However, how the OCSE-N affects their GNCI has not been studied. Therefore, we aimed to identify the potential characteristics of GNCI among geriatric nurses and explore the socio-demographic and clinical factors associated with different subgroups, with the aim of providing a theoretical basis and practical recommendations for enhancing the GNCI of different groups of geriatric nurses according to their characteristics.

## Methods

### Design

The study was a cross-sectional survey and adhered to the STROBE guideline for cross-sectional studies.

### Participants

In January-March 2024, a combination of stratified and whole cluster sampling was used to firstly divide the four official administrative districts of Hefei city(Yaohai district, Luyang district, Shushan district, and Baohai district) into basic units, and secondly, in each unit, two comprehensive medical institutions were randomly selected by means of the random number software, and one tertiary and one secondary hospitals were sampled, making a total of eight comprehensive medical institutions. Finally, all geriatric nurses who fulfilled the nerfing criteria were selected to participate in the questionnaire survey using whole cluster sampling in each healthcare institution. Inclusion criteria: (1) being a geriatric nurse; (2) having at least 1 year of work experience in geriatrics; (3) informed consent and voluntary participation in this study. Exclusion criteria: absentees such as those on sick and maternity leave, interns, and trainees.

### Sample size

The sample size was calculated as 10 times the number of items to be tested [22]. There are 69 items in this questionnaire. Therefore, the formula for sample size is *N*=(12 + 48 + 9) * 10 = 690, which means that at least 690 subjects are needed for this study. Also, the sample size should be further expanded considering the sample loss rate of 20%. Therefore, the minimum sample size required is *N* = 690÷(1–20%) ≈ 863.

### Data collection

A web-based survey was conducted using Questionstar. The Anhui Geriatric Nursing Alliance explained the purpose of the survey, the inclusion and exclusion criteria of the survey respondents to the nursing department and the head nurse of the geriatric department of the participating hospitals, and after obtaining informed consent, the head nurse of the geriatric department of the hospitals sent the QR code of Questionnaire Star through the WeChat platform to clinical nurses who met the criteria. Adopting a unified standard guideline and upholding the principles of informed consent, voluntariness, and non-harmfulness, the survey was filled out anonymously by the nurses to complete the survey independently and objectively. All questions of the questionnaire were set as mandatory, and each IP address could only fill in the questionnaire once. Data validation and entry were jointly completed by two researchers, and a total of 1400 questionnaires were distributed; However, data with a response time of less than 3 minutes (*n* = 47), questionnaires with obvious logical errors (*n* = 21), and questionnaires with a high degree of regularity (*n* = 19) were excluded, and 1,313 valid questionnaires were ultimately recovered, with an effective recovery rate of 93.8%.

### Instruments

#### General information questionnaire

The scale was compiled by this group on its own after referring to relevant and similar literature, and this study conducted a pre-survey before the formal survey, and the formal survey was conducted after the general information was supplemented and improved. It mainly includes 12 basic questions: age, gender, education, marital status, years of working experience, title, position, personnelnature, hospital grade, specialist nurse status, whether they have participated in geriatric specialist nurse training experience, and how much they like geriatric nursing.

#### Geriatric nursing competency inventory

The scale was developed in 2012 by the Chinese authors, Professor Wang Zhang’an et al. [6], by inviting 4 Thai and 10 Chinese professors in the field of geriatric nursing and 15 senior nurses who have long been engaged in geriatric nursing to participate in multiple rounds of expert correspondence, and was compiled by convenience sampling of 319 nurses from 32 long term care organisations in Nanning City, Guangxi Province, China, for the questionnaire’s reliability test, and the inclusion criteria of the nurses in this study were at least 1 year in geriatric nursing, secondary school or higher education in nursing, and whether or not they held a nursing licence were not required. The purpose of the study was to construct a scale to measure the core competencies of geriatric nursing suitable for China’s national conditions, with an overall Cronbach’s alpha coefficient of 0.980. The scale included critical thinking and research competencies (6 entries), clinical nursing competencies (12 entries), leadership (7 entries), interpersonal relationships(5 entries), legal and ethical qualities (7 entries), professional development and personal growth (4 entries), and educational/mentoring competencies (7 entries), for a total of 48 entries in 7 dimensions. A Likert 5-point scale was used, ranging from 0 to 4 on a scale from “not competent” to “very competent”, with a total score of 0 to 192, with higher scores indicating greater competence. The Cronbach’s alpha coefficient for the scale in this study was 0.991.

####  Occupational coping self-efficacy scale for nurse

The scale was compiled by Pisanti et al. [23] in 2008, and then by Zhai Yanxue et al. [24] in 2021 firstly by inviting two master’s degree in nursing to do the Chinese translation of the English questionnaire, then by a master’s degree in English (who had passed the eighth grade of the English profession) to do the English translation of the Chinese questionnaire, and then finally by a professor of nursing, a senior nurse working in a hospital ward, and a psychologist modified and rated the original and back-translated versions of the scale in accordance with the nature of the profession, the linguistic conventions, and the nurses’ ability to comprehend the scale, to obtain the Chinese version of the pilot scale. The scale was developed through a convenience sample of 1,172 nurses from five public hospitals in Shenzhen, Guangdong, China, who were tested for reliability and validity of the questionnaire, and the inclusion criteria for this study were inpatient nurses with a certificate of nursing practice. The purpose of the study was to construct a scale to measure nurses’ occupational coping self-efficacy suitable for the Chinese context, with an overall Cronbach’s alpha coefficient of 0.882. The Chinese version of the scale included occupational burdens (6 items) and relationship difficulties (3 items), with a total of 9 items in 2 dimensions. The scale was scored on a 5-point Likert scale ranging from 1 to 5 points from “unable to copeeasily” to “completely able to cope easily”, with a total score of 0 to 45 points, and the higher the score, the higher the nurses’ sense of efficacy in coping with their occupations. The Cronbach’s alpha coefficient of the scale in this study was 0.963.

### Data analysis

Data were analysed using SPSS 25.0 software, with measurements described by means and standard deviations, and counts described by frequencies and percentages, and comparisons between groups were made using the *χ*^*2*^ test or one-way ANOVA. Mplus 8.3 was used to analyse the latent category model forthe 48 entries of the GNCI. The model fitting indexes include: ①information index: Akaike Information Criterion (*AIC*), Bayesian Information Criterion (*BIC*), Adjusted Bayesian Information Criterion (*aBIC*), the smaller the value means the better the fitting effect; ②Classification index: *Entropy* value rangeis 0 ∼ 1, *Entropy* > 0.80 indicates that the classification accuracy is more than 90%, the closer to 1 indicates that the classification is more accurate; ③Likelihood ratio metrics: Likelihood ratio test metrics are corrected likelihood ratio test (*LMR*) and Bootstrap-based likelihood ratio test (*BLRT*), which indicates that a k-category model outperforms a k−1-category model when *P* < 0.05, requiring ≥ 5% sample size per profile [18]; The above evaluation indicators are only indicative and the interpretability of the categories should also be considered when determining the best model. After identifying the different categories of GNCI among geriatric nurses by LPA analysis, the variables with statistically significant differences between GNCI subgroups were first analysed by one-way analyses such as the *χ*^*2*^ test or one-way ANOVA (*P* < 0.05). Secondly, the above variables were included as independent variables in the multivariate logistic regression analysis with a test level of *α*=0.05.

### Ethical considerations

The study followed the ethical standards of the Declaration of Helsinki and was approved by the Ethics Committee of the First Affiliated Hospital of Anhui Medical University (PJ2024−06−31). Before distributing the questionnaires, we obtained informed consent from the participants with the assistance of the head nurse of the geriatric department in each hospital and obtained their handwritten and signed informed consent forms. Also all participants were informed that their participation was voluntary, that the questionnaire was anonymous and that they could withdraw from the study at any time without giving any reason. They were also assured that only the investigator could view the completed questionnaire and that this content would be used for the study only.

## Results

### Participant characteristics

A total of 1400 geriatric nurses were surveyed in this study and 1313 valid questionnaires were collected. The mean age of the respondents was 32.84 years (*SD* = 5.64; ranged from 24 to 50 years old) and more than half of the nurses were in the age group of 30 to 39 years (55.8%). There were 1202 females (91.5%), 903 married persons accounting for 68.8%, the largest proportion of nurses with 11 to 20 years of service (36.6%), with a predominantly bachelor’s degree (56.1%), more than half of the nurses had the title of charge nurse or above (55.5%), the vast majority of nurses (85.8%) did not hold concurrently an administrative position, and the largest proportion of nurses were employed through contractual arrangements (45.1%), more nurses from tertiary care hospitals (55.8), a smaller percentage of nurses (13.5%) had geriatric nurse specialist status, only 26.5% of geriatric nurses had participated in geriatric nurse specialist training, and nearly 85% of all nurses expressed a preference for geriatric nursing. Detailed demographics are shown in Table 1.

**Table 1:** Participants’ demography characteristics (*N* = 1313)

Variable	Number	Proportion (%)
Age (years)		
≤ 29	412	31.38
30 ∼ 39	733	55.83
≥ 40	168	12.79
Gender		
Female	1202	91.55
Male	111	8.45
Marital status		
Unmarried	394	30.00
Married	903	68.78
Divorced/widowed	16	1.22
Working years(years)		
1 ∼ 5	251	19.12
6 ∼ 10	471	35.87
11 ∼ 20	480	36.56
≥ 21	111	8.45
Education		
≤Junior college	429	32.67
Undergraduate	736	56.06
≥Graduate	148	11.27
Professional title		
Nurse	152	11.58
Senior nurse	432	32.90
≥Nurses–in–charge	729	55.52
Positions		
None	1126	85.76
Care team leader	54	4.11
≥Deputy chief nursing officer	133	10.13
Employment status		
Labor dispatching	592	45.09
Personnel agency	589	44.86
Aurhorized personnel	132	10.05
Specialist nurse status		
Non-specialist nurses	806	61.39
Other specialist nurses	330	25.13
Geriatric Nurse Specialist	177	13.48
Hospital level		
Tertiary hospital	733	55.83
Level II hospitals	580	44.17
Attendance at geriatric nurse specialist training		
Yes	348	26.50
No	965	73.50
Favouritism of elderly care		
Dislike	218	16.60
Generally preferred	662	50.42
Prefer	266	20.26
Favourite	167	12.72

### Descriptive statistics and correlations

There was a positive correlation between GNCI and OCSE-N of geriatric nurses. The GNCI score was 123.06(41.60) and the mean of the entries was 2.56(0.87) indicating that geriatric nurses’ GNCI scores were at an intermediate level. The OCSE-N scores were 35.44(7.34), which was at a relatively high level, as shown in Table 2.

**Table 2:** Descriptive statistics and correlation of GNCI, OCSE-N for geriatric nurses (*N* = 1313)

Items	M	SD	1	2	3	4	5	6	7	8	9	10	11
1 GNCI	123.06	41.60	1										
2 Critical thinking and scientific research skills	11.88	5.76	0.786*	1									
3 Clinical nursing competence	29.38	11.42	0.917*	0.777*	1								
4 Leadership	18.29	6.98	0.909*	0.653*	0.760*	1							
5 Interpersonal relationship	13.96	4.80	0.919*	0.604*	0.762*	0.883*	1						
6 Quality of legal ethics	20.69	6.83	0.866*	0.515*	0.679*	0.794*	0.879*	1					
7 Professional development and personal growth	10.62	3.85	0.916*	0.652*	0.777*	0.810*	0.847*	0.834*	1				
8 Educational/mentoring capacity	18.24	6.85	0.936*	0.669*	0.825*	0.820*	0.852*	0.807*	0.906*	1			
9 OCSE-N	35.44	7.34	0.620*	0.493*	0.556*	0.556*	0.576*	0.549*	0.583*	0.581*	1		
10 Occupational burden	23.31	5.01	0.612*	0.508*	0.560*	0.539*	0.555*	0.522*	0.575*	0.574*	0.987*	1	
11 Relationship difficulties	12.14	2.54	0.588*	0.425*	0.503*	0.545*	0.571*	0.559*	0.552*	0.550*	0.946*	0.880*	1

Note.*M* = mean;*SD* = standard deviation; *P < 0.05

### Exploratory latent profle analysis

**Fig. 1:**
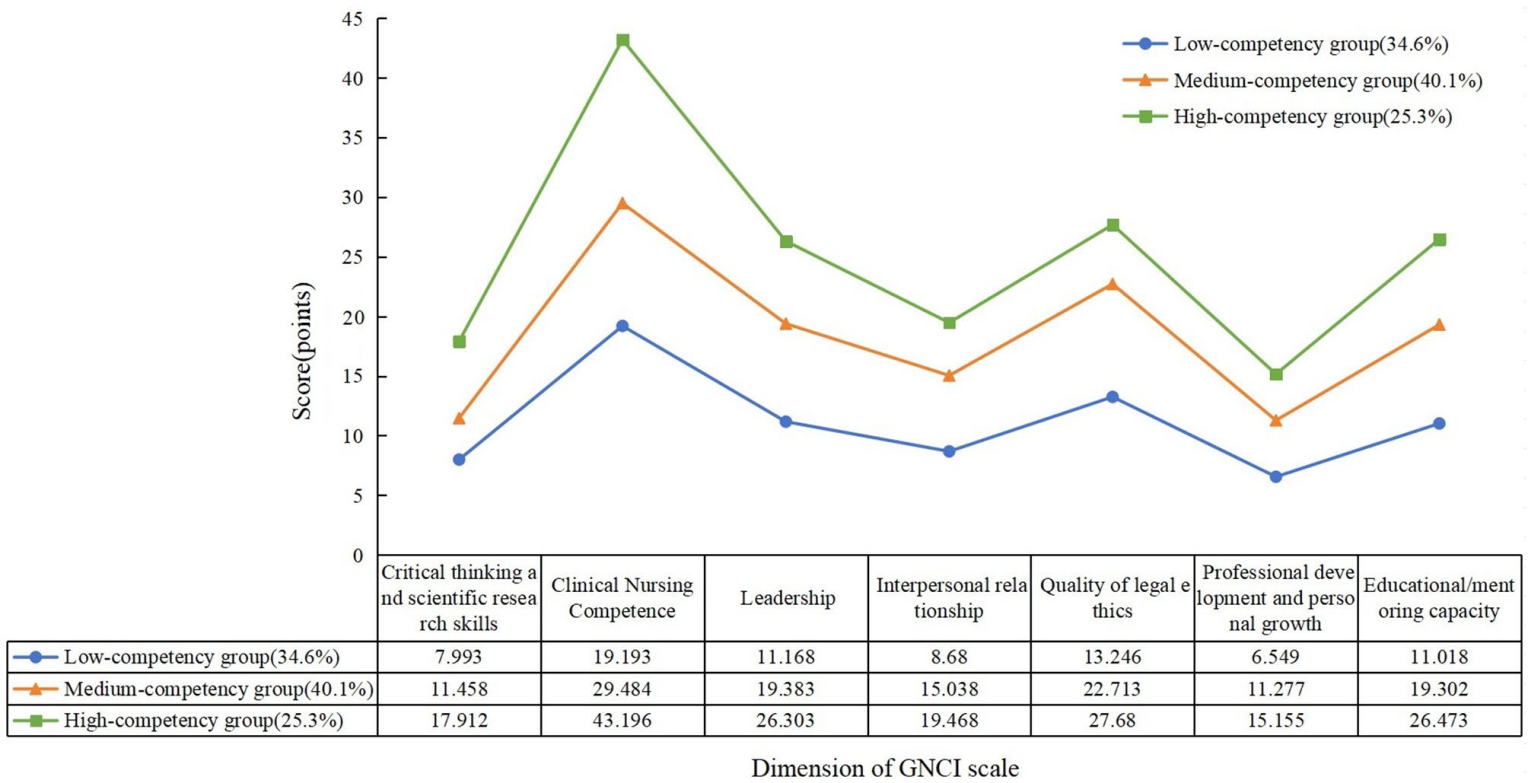
Geriatric nurses had different levels of GNCI. Category 1: Low-competency group; Category 2: Medium-competency group; Category 3: High-competency group

In this study, the seven dimensions of GNCI were used as the exogenous indicators to fit and analyse the potential profile models of categories 1 to 6, as shown in Table 3. The values of *AIC*, *BIC*, and *aBIC* gradually decreased with the increase of the number of categories until the *LMR* indicators of model 6 were not statistically significant (*P* > 0.05), which suggests that the fitting effect is getting better and better with the increase of the number of categories in the model 1 to model 5. The *Entropy* values of Model 2 to Model 5 are all greater than 0.8, indicating that the classification accuracy of the model is more than 90% in all cases, with Model 4 having the largest *Entropy* value, which indicates that it has the highest classification accuracy. The *P*-values of *LMRT* and *BLRT* from Model 2 to Model 5 are all < 0.05, indicating that the latter category models are all better than the former category models. However, the proportion of the sample size of individual groups in Models 4 and 5 did not exceed 10%, and the total sample size varied greatly between groups. Model 3 has ideal fit evaluation indexes and sample sizes for each group, and the probabilities of belonging to the three potential categories are 0.982, 0.972, and 0.970, respectively, indicating that the model is reliable for categorisation, and the results are shown in Table 4. Considering the interpretability of each category, and the practical significance of the categorisation, model 3 is finally selected as the best-fitting model, and is shown in Fig. 1. In the 3-category model, category 1, with a total of 455 (34.6%) nurses, had the lowest scores on all dimensions and was named the “Low-competency group”, with a score of 77.92(21.91), which is represented by C1. Category 2 consisted of 526 (40.1%) nurses with moderate scores on each dimension and was named the “Medium-competency group” with a score of 128.60(14.83), which is represented by C2. Category 3 has 332 (25.3%) nurses with high scores in all dimensions, including the highest score in the dimension of clinical nursing competence, which indicates that this category of nurses can accurately grasp the characteristics of the illness of the elderly patients in clinical work, identify the needs of the patients and their families, and give personalised and targeted nursing measures in a timely manner, and so it is named as the “High-competency group”, with a score of 176.17(13.15), which is indicated by C3. The results are shown in Fig. 1.

**Table 3:** Geriatric nurse GNCI potential profile model fit information (*N* = 1313)

Classes	AIC	BIC	aBIC	Entropy	LMR	BLRT	Categorical probability(%)
1	59954.968	60027.489	59983.018	-	-	-	-
2	54027.062	54141.023	54071.139	0.956	< 0.001	< 0.001	0.434/0.566
**3**	**51754.461**	**51909.863**	**51814.567**	**0.946**	**< 0.001**	**< 0.001**	**0.346/0.401/0.253**
4	49585.447	49782.290	49661.582	0.962	< 0.001	< 0.001	0.107/0.349/0.309/0.235
5	49164.414	49402.698	49256.577	0.947	0.0229	< 0.001	0.104/0.234/0.099/0.328/0.235
6	48694.855	48974.579	48803.046	0.949	0.0503	< 0.001	0.098/0.224/0.106/0.235/0.122/0.215

Note. Bold values indicate the optimal model; Abbreviations:*AIC* Akaike Information Criterion;*BIC* Bayesian Information Criterion;*aBIC* Adjusted *BIC*;*LMR* Lo-Mendell‐Rubin Test;*BLRT* Bootstrap Likelihood Ratio Test;—Not applicable

**Table 4:** Geriatric nurse GNCI potential profile category attribution probability matrix (%)

Potential profile type	Probability of belonging to a potential category		
	C1	C2	C3
C1	0.982	0.018	<0.001
C2	0.012	0.972	0.015
C3	<0.001	0.030	0.970

Note. Category 1: C1Low-competency group; Category 2: C2Medium-competency group; Category 3: C3High-competency group

### Characteristics of latent profle membership

The results of univariate analysis showed that nurses with different potential profiles were distributed differently in terms of years of experience, education, title, position, specialist nurse status, whether they participated in gerontological specialist nurse training, and scores of liking for geriatric care, and the differences were statistically significant (*P* < 0.05). The scores of each dimension of GNCI for nurses of group C1 were significantly lower than those of the overall level. The main characteristics of the nurses in the Low-competency group were, no position, non-specialist nurses, and did not participate in geriatric specialist nurse training. Group C3 had the highest scores for each dimension, and this group consisted mainly of nurses with nurses–in–charge nurse or higher, with specialist nurse status, and participated in geriatric specialist nurse training. The patients in group C2, who did not show any significant characteristics in their general demographic information, had a level of the GNCI that fell in-between, which could be facilitated by appropriate guidance and organisational strategies to convert them to the C3 group. In addition, the High-competency group scored significantly higher in positive coping with occupational burden and difficulties in getting along in relationships compared with the other categories, as detailed in Table 5.

**Table 5:** Differences in GNCI latent traits among geriatric nurses in terms of demographics, and nurse occupational coping self-efficacy (*N* = 1313)

Variable	Groups			χ^2^/F	*P*
	Low-competency group	Medium-competency group	High-competency group		
Age (years)				8.774	0.067
≤ 29	165(36.3)	158(30.0)	89(26.8)		
30 ∼ 39	235(51.6)	299(56.9)	199(59.9)		
≥ 40	55(12.1)	69(13.1)	44(13.3)		
Gender				2.966	0.227
Female	411(90.3)	490(93.2)	301(90.7)		
Male	44(9.7)	36(6.8)	31(9.3)		
Marital status				3.695	0.449
Unmarried	138(30.3)	155(29.5)	101(30.4)		
Married	309(67.9)	368(70.0)	226(68.1)		
Divorced/widowed	8(1.8)	3(0.5)	5(1.5)		
Working years(years)				17.348	0.008^*^
1 ∼ 5	90(19.8)	104(19.8)	57(17.2)		
6 ∼ 10	161(35.4)	192(36.5)	118(35.5)		
11 ∼ 20	182(40.0)	183(34.8)	115(34.6)		
≥ 21	22(4.8)	47(8.9)	42(12.7)		
Education				39.566	< 0.001^*^
≤Junior college	173(38.1)	184(35.0)	72(21.7)		
Undergraduate	250(54.9)	286(54.4)	200(60.2)		
≥Graduate	32(7.0)	56(10.6)	60(18.1)		
Professional title				23.670	< 0.001^*^
Nurse	64(14.1)	60(11.4)	28(8.4)		
Senior nurse	178(39.1)	158(30.0)	96(28.9)		
≥Nurses–in–charge	213(46.8)	308(58.6)	208(62.7)		
Positions				25.939	< 0.001^*^
None	416(91.4)	448(85.2)	262(78.9)		
Care team leader	14(3.1)	22(4.2)	18(5.4)		
≥Deputy chief nursing officer	25(5.5)	56(10.6)	52(15.7)		
Employment status				4.829	0.305
Labor dispatching	205(45.1)	242(46.0)	145(43.7)		
Personnel agency	205(45.1)	223(42.4)	161(48.5)		
Aurhorized personnel	45(9.8)	61(11.6)	26(7.8)		
Specialist nurse status				171.024	< 0.001^*^
Non-specialist nurses	337(74.1)	363(69.0)	106(31.9)		
Other specialist nurses	83(18.2)	111(21.1)	136(41.0)		
Geriatric Nurse Specialist	35(7.7)	52(9.9)	90(27.1)		
Hospital level				4.634	0.099
Tertiary hospital	268(58.9)	295(56.1)	170(51.2)		
Level II hospitals	187(41.1)	231(43.9)	162(48.8)		
Attendance at geriatric nurse specialist training				66.123	< 0.001^*^
Yes	79(17.4)	127(24.1)	142(42.8)		
No	376(82.6)	399(75.9)	190(57.2)		
Favouritism of elderly care				133.039	< 0.001^*^
Dislike	106(23.3)	87(16.5)	25(7.5)		
Generally preferred	261(57.4)	278(52.9)	123(37.1)		
Prefer	59(13.0)	108(20.5)	99(29.8)		
Favourite	29(6.3)	53(10.1)	85(25.6)		
Nurses’ occupational coping self-efficacy					
Occupational burden	19.96 ± 4.90	23.67 ± 3.99	27.33 ± 3.11	307.413	< 0.001^*^
Relationship difficulties	10.38 ± 2.57	12.45 ± 1.94	14.04 ± 1.58	299.677	< 0.001^*^

Note.*Significant at the 0.05 level

### Predictors of latent profle membership

We performed a multinomial logistic regression to verify the influence of GNCI in the three latent characteristics, using the Low-competency group as a reference, and the results are shown in Table 6. There were no differences between profiles in terms of years of experience, education, job title, and liking for elderly care among nurses. The membership characteristics of C1 (“usually low levels of GNCI and insufficient levels of coping in terms of clinical knowledge, skills, and interpersonal relationships”) were higher than the membership characteristics of C2 (“Medium-competency group”) and C3 (“High-competency group with high levels of clinical knowledge skill acquisition, interpersonal coping and self-development”) members with lower levels of OCSE-N. In addition, members of C2 (“Medium- competency group”) had lower OCSE-N levels than members of C3 (“High-competency group with high levels of clinical knowledge and skill acquisition, interpersonal relationship management and self-development”). In terms of job title and specialist nurse status, the characteristics of members of C1 (“usually have low levels of GNCI and inadequate levels of clinical knowledge, skills and interpersonal coping”) were lower than those of members of C2 (“Medium-competency group”) and C3 (“High-competency group with high levels of of clinical knowledge and skills acquisition, interpersonal coping and self-development”) members with low job titles and a high proportion of non-specialist nurses. Members who participated in geriatric nurse specialist training were more likely to be in C2 (“Medium-competency group”) and C3 (“High-competency group with high levels of clinical knowledge and skills acquisition, interpersonal skills and self-development”) than in C1 (“usually low levels of GNCI and insufficient levels of coping in terms of clinical knowledge, skills, and interpersonal relationships”).

**Table 6:** The multifactor analysis of geriatric nurses’ GNCI by logistic regression(*N* = 1313)

Variable	β	Wald χ^2^	*P*	OR	95%CI
**C2 vs. C1** ^**1)**^					
Intercept	-3.297	23.914	< 0.001	-	-
Nurses’ occupational coping self-efficacy	0.135	122.644	< 0.001*	1.145	(1.118,1.173)
Working years(years)					
1 ∼ 5	-0.227	0.446	0.504	0.797	(0.410,1.550)
6 ∼ 10	-0.318	1.006	0.316	0.727	(0.390,1.355)
11 ∼ 20	-0.499	2.515	0.113	0.607	(0.328,1.125)
≥ 21 (refer)					
Education					
≤Junior college	-0.331	1.369	0.242	0.718	(0.412,1.251)
Undergraduate	-0.395	2.139	0.144	0.674	(0.397,1.144)
≥Graduate (refer)					
Professional title					
Nurse	-0.327	2.031	0.154	0.721	(0.460,1.130)
Senior nurse	-0.416	6.665	0.010*	0.659	(0.481,0.905)
≥Nurses–in–charge (refer)					
Positions					
None	-0.540	3.549	0.060	0.583	(0.332,1.022)
Care team leader	-0.352	0.571	0.450	0.703	(0.282,1.754)
≥Deputy chief nursing officer (refer)					
Attendance at geriatric nurse specialist training					
Yes	0.210	1.348	0.246	1.234	(0.865,1.759)
No (refer)					
Specialist nurse status					
Non-specialist nurses	0.188	0.429	0.512	1.207	(0.687,2.122)
Other specialist nurses	0.182	0.356	0.551	1.200	(0.660,2.183)
Geriatric Nurse Specialist (refer)					
Favouritism of elderly care					
Dislike	-0.102	0.099	0.754	0.903	(0.478,1.705)
Generally preferred	0.026	0.008	0.930	1.026	(0.573,1.839)
Prefer	0.381	1.396	0.237	1.464	(0.778,2.757)
Favourite (refer)					
**C3 vs. C1** ^**1**)^					
Intercept	-10.714	113.087	< 0.001	-	-
Nurses’ occupational coping self-efficacy	0.321	246.245	< 0.001*	1.378	(1.324,1.435)
Working years(years)					
1 ∼ 5	0.385	0.845	0.358	1.469	(0.647,3.336)
6 ∼ 10	0.027	0.005	0.944	1.027	(0.487,2.165)
11 ∼ 20	-0.290	0.595	0.440	0.748	(0.358,1.564)
≥ 21 (refer)					
Education					
≤Junior college	-0.627	3.115	0.078	0.534	(0.266,1.072)
Undergraduate	-0.417	1.668	0.196	0.659	(0.350,1.241)
≥Graduate (refer)					
Professional title					
Nurse	-0.754	5.074	0.024*	0.470	(0.244,0.907)
Senior nurse	-0.560	6.447	0.011*	0.571	(0.371,0.880)
≥Nurses–in–charge (refer)					
Positions					
None	-0.490	2.029	0.154	0.613	(0.312,1.202)
Care team leader	-0.566	0.995	0.319	0.568	(0.187,1.727)
≥Deputy chief nursing officer (refer)					
Attendance at geriatric nurse specialist training					
Yes	0.767	11.858	0.001*	2.154	(1.392,3.333)
No (refer)					
Specialist nurse status					
Non-specialist nurses	-0.996	9.225	0.002*	0.369	(0.194,0.702)
Other specialist nurses	0.228	0.471	0.493	1.255	(0.656,2.405)
Geriatric Nurse Specialist (refer)					
Favouritism of elderly care					
Dislike	-0.535	1.761	0.185	0.586	(0.266,1.291)
Generally preferred	-0.039	0.014	0.905	0.962	(0.504,1.836)
Prefer	0.558	2.490	0.115	1.748	(0.874,3.498)
Favourite (refer)					
**C2 vs. C3** ^**2**)^					
Intercept	7.417	71.406	< 0.001	-	-
Nurses’ occupational coping self-efficacy	-0.185	104.837	<0.001*	0.831	(0.802,0.861)
Working years(years)					
1 ∼ 5	-0.611	3.462	0.063	0.543	(0.285,1.033)
6 ∼ 10	-0.345	1.415	0.234	0.708	(0.401,1.251)
11 ∼ 20	-0.209	0.530	0.467	0.811	(0.462,1.424)
≥ 21 (refer)					
Education					
≤Junior college	0.296	1.095	0.295	1.344	(0.772,2.339)
Undergraduate	0.022	0.008	0.928	1.023	(0.630,1.660)
≥Graduate (refer)					
Professional title					
Nurse	0.428	2.057	0.152	1.534	(0.855,2.752)
Senior nurse	0.143	0.547	0.460	1.154	(0.789,1.687)
≥Nurses–in–charge (refer)					
Positions					
None	-0.050	0.037	0.847	0.951	(0.570,1.586)
Care team leader	0.213	0.242	0.623	1.238	(0.529,2.897)
≥Deputy chief nursing officer (refer)					
Attendance at geriatric nurse specialist training					
Yes	-0.557	9.272	0.002*	0.573	(0.400,0.820)
No (refer)					
Specialist nurse status					
Non-specialist nurses	1.184	20.908	<0.001*	3.269	(1.967,5.431)
Other specialist nurses	-0.045	0.033	0.856	0.956	(0.585,1.562)
Geriatric Nurse Specialist (refer)					
Favouritism of elderly care					
Dislike	0.433	1.689	0.194	1.542	(0.802,2.962)
Generally preferred	0.065	0.069	0.793	1.068	(0.656,1.738)
Prefer	-0.177	0.473	0.492	0.838	(0.506,1.387)
Favourite (refer)					

Note. *Refer* Reference group; *Significant at the 0.05 level; ^1)^Low-competency group profile as the reference category; ^2)^High-competency group profile as the reference category

## Discussions

### Potential characteristics of the GNCI in geriatric nurses and their application tonursing practice

Through latent trait analysis, this study found that the GNCI of geriatric nurses can be divided into three characteristics: three “C1 Low-competency group”, “C2 Medium-competency group” and “C3 High-competency group” categories, suggesting significant individual differences in the GNCI of geriatric nurses. C2 had the highest number (40.1%), C1 was the low ability group (34.6%), and the C3 group had the lowest number (25.3%). This result suggests that the GNCI of geriatric nurses is at a moderately level, which is consistent with the findings of Wang et al [25]. but lower than those of Chunlan et al. [26]. The reason for this analysis was that the subjects of Chunlan et al.‘s study were all geriatric nurses specialist, who received professional theory and practice courses in geriatric nursing ranging from 1 to 12 months, and were able to accurately assess the changes in patients’ conditions, meet patients’ needs, and participate more in the application of gerontological nursing best practices, so their core competencies were higher. Saliaet al. also showed that geriatric nursing training promotes nurses to acquire systematic and sound theoretical knowledge and skilled nursing techniques, so that they are better able to cope with complex work situations and improve nursing competence and patient outcome [27]. Gu and other researchers have shown that a Six Sigma training programme that takes into full consideration the actual needs of nurses, their satisfaction with participation and the goal of improving their core competencies, strictly controls the quality of base management, theoretical and practical teaching, and regularly collects the opinions of nurses and trainers for timely reflection and continual improvement can strengthen nurses’ basic management skills, broaden their interest in the theoretical courses and expand the breadth, depth and practicability of the courses, and then improve their core competencies [28]. In addition, diversified training and teaching methods, such as virtual reality (VR) teaching, simulated ward teaching, and case studies, not only meet the daily training needs of nurses, but also enhance their positive learning attitudes and improve learning efficiency and satisfaction [29, 30]. This suggests that nursing managers should not only pay attention to and increase the GNCI training of geriatric nurses, but more importantly pay attention to the needs of nurses to be trained, and constantly update the teaching and training methods and forms, to enhance the internalisation and absorption of their knowledge, and thus enhance the GNCI.

Nurses in group C1 scored significantly lower on all dimensions of the GNCI than the overall group, and as can be seen in Table 5, nurses in group C1 had a greater proportion of nurses with no duties, non-specialist nurses, and no geriatric nurse specialist training. This indicates that the nurses in this group were more influenced by the constraints of their administrative duties and teaching training. The nurses in this group had not been trained in professional and systematic nursing knowledge, lacked rich geriatric nursing knowledge and skills, and had not yet formed a system and process for the management of elderly patients and the handling of emergencies, which weakened the intrinsic motivation and confidence in geriatric nursing and led to a low GNCI [27, 31]. At the same time, the nurses in this group had fewer administrative duties, mainly undertook basic nursing care in the high-stress, high-stress geriatric nursing work environment, with less clinical risk and a lower sense of responsibility for patients, making it difficult for them to provide more humanistic care for patients in a mobile and flexible manner while completing routine treatment. This suggests that medical administrators should focus on strengthening the training of C1 group nurses’ knowledge and skills related to core competencies in geriatric nursing, which can draw on the competency progression hierarchical training programme to develop a training programme to enhance the group’s theoretical knowledge and job competency based on the hierarchical division of nurses’ positions and different training needs, in order to enhance and improve the level of nurses’ GNCI [32].

The nurses in group C2 were the current leading practitioners of clinical geriatric nursing with extensive experience in clinical practice and a high level of knowledge and skills related to geriatric nursing. The nurses in this group did not show significant characteristics in their general demographic profile, and it is worth noting that critical thinking and research skills scored the lowest compared to the scores of the nurses in group C2 on the seven dimensions (Fig. 1). Analysing the content of the entries corresponding to this dimension, we found that the nurses in this group were less sensitive to the identification of clinical problems, cutting-edge nursing knowledge and research hotspots, and were reluctant to take the initiative in accepting and challenging new nursing knowledge and technology and actively applying them in the clinic in order to enhance their strengths, thus having a higher level of GNCI and execution ability. This may be related to the fact that very few nurses in this group have postgraduate qualifications, and the vast majority of nurses have not received professional and systematic research training [33]. It is recommended that future training programmes should include master’s degree training courses or rely on special educational tools such as knowledge mapping to provide nurses with refined teaching resources, and that continuing education should be enhanced to make up for the shortcomings of the current training [34].

Nurses in group C3 had the highest scores in all dimensions of GNCI, and this group mainly consisted of nurses with the title of nurses–in–charge and above, with the status of specialist nurses, and participated in geriatric specialist nurse training. Nurses in this group generally have longer working years, are experienced in their own experience and work, and have received training and assessment of professional knowledge and skills, so their knowledge and understanding of geriatric nursing work is clearer and their work content is more familiar, so their GNCI is high [27, 31]. It is recommended that managers should give full play to the leading role of nurses in this group and encourage teamwork between senior and young nurses in order to promote the overall improvement of GNCI among geriatric nurses.

### Impact of demographic characteristics on the GNCI of geriatric nurses

The results of this study showed that nurses with the title of nurse and senior nurse had a greater probability of entering the C1 group compared to those with the title of nurses–in–charge and above, suggesting that nurses with lower titles had a lower GNCI, which is similar to the results of Zhou et al [26, 35, 36]. It may be related to the fact that nurses with lower job titles have entered the workforce for a shorter period of time and are less experienced in their work, and their competence in various aspects has not yet been effectively upgraded [37]. Baek et al. showed that enhancing nursing teamwork is an effective strategy for promoting quality of care. This suggests that healthcare managers can actively implement organisational strategies such as selecting highly qualified and experienced nurses and adopting a ‘helping and leading’ model of collaboration between the old and the new to support teamwork in nursing and thereby improve the overall quality of geriatric care [38]. In addition, Wang et al. showed that when a nurse’s title reaches the intermediate level, his/her core competitiveness is in a period of rapid rise, in which nursing managers should fully provide nurses with a platform for demonstration and development, so as to match nurses’ competence with their positions, satisfy the need for self-fulfilment, and improve nurses’ sense of professional achievement, which will in turn enhance the level of GNCI [39].

Nurses who had participated in geriatric nurse specialist training were more likely to enter the C3 group, suggesting that active participation in geriatric nurse specialist training promotes nurse GNCI, which is in line with Chen et al. [17]. It may be that the training will stimulate nurses’ enthusiasm and cohesionfor geriatric care, enhance their geriatric knowledge and skills, and thus continue to improve their core competencies in meeting the health needs of older people [40]. However, this result is inconsistent with the findings of Chen et al [41]. The results of Chen’s study showed that 70.6% of the nurses had participated in geriatric nursing-related training before joining the nursing facility but it had no effect on the level of GNCI, which may be related to the differencesin the training methods between the nursing facility and the general hospital. A study suggests that only a uniform training programme and admission requirements for geriatric nurses of at least three months or more in professional knowledge, technology and research skills is more likely to achieve the desired training outcomes and meet clinical care needs [42]. It is recommended that medical managers should not only expand their training efforts, but also set strict training standards, effective training hours and organise regular theoretical and practical assessments after the course, and activities such as competitions and sharing sessions for practical results are also essential.

Nurses with non-specialist nurse status were more likely to be in groups C1and C2, which suggests that geriatric specialist nurses have a relatively high GNCI, which is similar to the findings of Yang et al. [43]. Possible reasons for this are as follows: firstly, geriatric nurse specialist are assessed on the basis of established criteria, including years of experience, teaching experience, theoretical and skills training and assessment, which suggests that geriatric nurse specialist are somehow more competent than others and have more opportunities and learning resources to continue to develop their knowledge and skills [40]. Secondly, having specialist nurse status means that the greater the responsibility and pressure of the job, the more self-restraint he or she will have, the more eager he or she will be to improve himself or herself, and the higher his or her expectations of responsibility will be, thus forcing geriatric nurse specialist to continually improve their GNCI [44]. Although the GNCI of geriatric nurse specialist is on the high side, the greater stress brought about by their job duties also requires constant attention and confrontation. Wang et al. showed that a positive thinking intervention through guidance that allowed participants to learn to be purposefully alert and attentive to the present moment and to self-observe in an objective and transcendent manner not only effectively reduced nurses’ common psychological symptoms such as depression, anxiety, and stress, but also, by increasing present-moment awareness, could enhance nurses’ attention, concentration and ability to cope with problems, which in turn promotes geriatric nurses to provide high-quality nursing services to patients with a more confident attitude [45, 46]. This suggests that nursing managers should not only pay more attention to the GNCI enhancement of geriatric nurses and encourage their active participation in geriatric specialist training programmes, but also positive psychological guidance and support should not be overlooked.

The results of this study showed that OCSE-N was a significant predictor of GNCI for geriatric nurses, and geriatric nurses’ OCSE-N was positively correlated with GNCI, and nurses with a high level of OCSE-N were more likely to fall into the C2 and C3 groups, suggesting that geriatric nurses with a positive OCSE-N had higher GNCI, similar to the results of the study by Chen et al. [47, 48]. This may be related to the fact that geriatric nurses with high OCSE-N have a clear clinical work plan, are better able to face and deal with occupational burdens, relationship difficulties and other complex clinical problems, and are more likely to take the lead in overcoming challenges with positive emotions, which enhances their enthusiasm and initiative, and thus contributes to the improvement of GNCI [49–51]. In addition, nurses with low OCSE-N are less willing to participate in projects that require high GNCIs and are prone to give up when they encounter obstacles and problems, which ultimately leads to ineffective enhancement of core competencies, further exacerbating the fearfulness and creating a vicious cycle [52, 53]. Research has shown that managers with an empowering leadership style will motivate their employees to move forward through visionary inspiration, affirmation of competence, and participation in decision-making, so that they can effectively deal with and overcome difficultiesat work, and feel confident and energised about the prospects and development of their careers [54]. However, although empowering leadership style can promote positive behaviours of employees, if the strength of leadership empowerment exceeds a certain amount, it will increase the workload of employees, consume their positive emotions, make them unable to concentrate on coping with complicated work, and significantly reduce their work motivation and initiative [55]. This suggests that nursing managers should reasonably allocate work tasks, do a good job of integrating and deploying human resources, and at the same time do a good job of personalised career development planning guidance in order to enhance the effectiveness of nurses’ career coping and strengthen their core competencies.

## Conclusions

There is heterogeneity in the GNCI category of geriatric nurses, which can be classified into 3 categories i.e. Low-competency group, Medium-competency group and High-competency. Geriatric nurses with low OCSE-N, nurse and senior nurse, who did not participate in geriatric nurse speciality training, and non-specialist nurse status had low GNCI. Nursing managers should pay attention to the differences in nurses’ GNCI and carry out targeted and varied management strategies based on the profiles of different types of groups to improve geriatric nurses’ OCSE-N and GNCI, to ensure the quality of geriatric nursing care, to reduce nursing turnover, and to alleviate nursing shortages.

## Data Availability

The data supporting the fndings of this study are available on request from the corresponding author. The data are not publicly available due to privacy orethical restrictions.

## References

[1] United Nations Department of Economic and Social Affairs. Population division. World Population Prospects. 2022. https://population.un.org/wpp/. Accessed 17 Apr 2024.

[2] Chen X, Giles J, Yao Y, et al. The path to healthy ageing in China: a Peking University-Lancet Commission. Lancet. 2022;400(10367):1967–2006. 36423650 10.1016/S0140-6736(22)01546-X PMC9801271

[3] Centers for Disease Control and Prevention. (2019). Persons with hospital stays in thepast year, by selected characteristics: United States, selected years 1997–2018. https://www.cdc.gov/nchs/data/hus/2019/040-508.pdf. Accessed 15 Apr 2024.

[4] Buerhaus PI, Chang Y, DesRoches C, et al. The roles and clinical activities of registered nurses and nurse practitioners in practices caring for older adults. Nurs Outlook. 2021;69(03):380–8. 33422289 10.1016/j.outlook.2020.11.011

[5] Hu FW, Lee HF, Li YP. Exploration of geriatric care competencies in registered nurses in hospitals. J Nurs Res. 2021;29(04):e159. 34034305 10.1097/JNR.0000000000000441

[6] Wang Z. A qualitative study of the construction of indicators for measuring core competencies in geriatric care. Chin J Nurs. 2012;47(05):457–9.

[7] National Health Commission. Notice of the General Office of the National Health Commission on the Pilot Work of Medical Care Services for the Elderly. 2021. http://ww.gov.cn/zhengce/zhengceku/2021-11/27/content_5653768.htm. Accessed 29 Jun 2024.

[8] Chen J, Zhang N, Guo JJ, et al. Survey on the demand for geriatric nursing core competencies among elderly groups in medical and nursing care organisations[J]. Nurs Res. 2019,33(08):1385–9.

[9] Kiljunen O, Välimäki T, Kankkunen P, et al. Competence for older people nursing in care and nursing homes: an integrative review. Int J Older People Nurs. 2017;12(03):10. 10.1111/opn.12146 28032436

[10] Lin XJ, Chen S. Observation on the effect of modular teaching method oriented to core competence of geriatric nursing in the teaching of geriatric nursing students[J]. China High Med Educ. 2023;(10):107–8.

[11] Yan XJ. Construction of the training programme for community specialist nurses basedon core competence-taking the direction of geriatric nursing as an example[D]. Jiangsu University; 2023.

[12] Yao XY, Zhang HH, Xing Q. The construction of the training target system of geriatric advanced clinical nurse specialists in China[J]. Nurs Res. 2022;36(18):3270–6.

[13] Su YT. Construction of core competence evaluation index system for elderly caregivers in medical and nursing institutions[D]. China Medical University; 2023.

[14] Tang XT. Research on the competence of nurses’ geriatric health service in primary healthcare institutions in the context of active ageing[D]. Shihezi University; 2024.

[15] Ferreira R, Derhun FM, Carreira L, et al. Professional competency for elder care: perception among professors, nursing students, and nurses. Rev Bras Enferm. 2021;74Suppl(02):e20200446. 10.1590/0034-7167-2020-0446 33787810

[16] Chen H, Pu L, Chen Q, et al. Instrument development for evaluation of gerontological nurse specialists core competencies in China. Clin Nurse Spec. 2019;33(05):217–27. 31404000 10.1097/NUR.0000000000000469

[17] Chen H, Pu L, He S, et al. Status and associated factors of gerontological nurse specialists’ core competency: a national cross-sectional study. BMC Geriatr. 2023;23(01):45. 37479983 10.1186/s12877-023-04153-0 PMC10362742

[18] Tein JY, Coxe S, Cham H. Statistical power to detect the correct number of classesin latent profile analysis. Struct Equ Model. 2013;20(04):640–657. 10.1080/10705511.2013.824781 PMC3904803 24489457

[19] Fida R, Laschinger HKS, Leiter MP. The protective role of self-efficacy against workplace incivility and burnout in nursing: a time-lagged study. Health Care Manage Rev. 2018;43(01):21–29. 27755174 10.1097/HMR.0000000000000126

[20] Bandura A. Social cognitive theory of self-regulation. Organ Behav Hum Decis Process. 1991;50(02):248–87. 10.1016/0749-5978(91)90022-L

[21] Wu J, Li Y, Lin Q, et al. The effect of occupational coping self-efficacy on presenteeism among ICU nurses in Chinese public hospitals: a cross-sectional study. Front Psychol. 2024;15:1347249. 38356774 10.3389/fpsyg.2024.1347249 PMC10865889

[22] Wu C, et al. Patterns of information literacy and their predictors among emergency department nurses: a latent profile analysis based on the person-context interaction theory. BMC Nurs. 2024;23(01):71. 38279169 10.1186/s12912-024-01756-9 PMC10811938

[23] Pisanti R, Lombardo C, Lucidi F, et al. Development and validation of a brief occupational coping self-efficacy questionnaire for nurses. J Adv Nurs. 2008;62(02):238–47. 18394036 10.1111/j.1365-2648.2007.04582.x

[24] Zhai Y, Chai X, Liu K, et al. A study on the sinicisation and reliability of the nurses’ occupational coping self-efficacy scale. Mod Prev Med. 2021;48(03):423–6.

[25] Wang Y, Fan J, Wang M, et al. Analysis of the current status and influencing factors of geriatric nursing core competencies of nurses in Suzhou nursing homes. Health Career Educ. 2022;40(14):114–7.

[26] Chunlan B, Lihui P, Hongxiu C, et al. The gerontological nurse specialist’s core competencies in China: a cross-sectional study. Nurs Open. 2020;7(06):1928–35. 33072378 10.1002/nop2.583 PMC7544875

[27] Salia SM, Adatara P, Afaya A, et al. Factors affecting care of elderly patients among nursing staff at the Ho teaching hospital in Ghana: implications for geriatric care policy in Ghana. Plos One. 2022;17(06):e0268941. 35737704 10.1371/journal.pone.0268941 PMC9223345

[28] Gu J, Luo L, Li C, Ma S, Gong F. Effects of a modified six-sigma-methodology-based training program on core competencies in rehabilitation nurse specialists. J Korean Acad Nurs. 2023;53(04):412–5. 37673816 10.4040/jkan.22122

[29] Xie Z, Chen F, Zou L, et al. Using virtual reality in the care of older adults with dementia: a randomized controlled trial. J Gerontol Nurs. 2023;49(11):25–32. 37906042 10.3928/00989134-20231011-01

[30] Hsieh PY, Lin HY, Chang CH, et al. Effects of situational simulation and online first-aid training programs for nurses in general medical wards: a prospective study. Nurse Educ Today. 2021;96:104621. 33197681 10.1016/j.nedt.2020.104621

[31] Luo H, Gong H, Luo F, et al. Core competence of midwives in township hospitals and its influencing factors-A cross-sectional study. Heliyon. 2024;10(03):e25475. 38327397 10.1016/j.heliyon.2024.e25475 PMC10848002

[32] Zhang X, Yuan X, Li J, et al. The effect of competency progression stratification training based on Benner’s theory on the comprehensive ability and job competency of nurses in primary hospitals. Nurs Res. 2021;35(23):4278–4281.

[33] Li DP, Zhou XM, Zhang ZH, et al. Survey on the current status of core competence of 215 intravenous therapy nurses in Guangdong Province and analysis of influencing factors[J]. J Nurse Advancement. 2021;36(23):2160–6.

[34] Liang QK, Gao J, Tan YB. A qualitative study of facilitators and impediments to job competency enhancement among speciality nurses[J]. J Nurs. 2024;39(09):73–6.

[35] Zhou AJ, Jiang LP. The current situation of core competence of nurses and its influencing factors in nursing institutions in Jinhua City[J]. Journal of PLA Nursing. 2015;32(15):63–5.

[36] Feng P, Hao J, Wang Y, et al. A cross-sectional survey on nurses in burn departments: core competencies and influencing factors. Burns. 2023;49(05):1218–24. 36195481 10.1016/j.burns.2022.09.003

[37] Wei W, Niu Y, Ge X. Core competencies for nurses in Chinese intensive care units: a cross-sectional study. Nurs Crit Care. 2019;24(05):276–82. 30569548 10.1111/nicc.12398

[38] Baek H, Han K, Cho H, et al. Nursing teamwork is essential in promoting patient-centered care: a cross-sectional study. BMC Nurs. 2023;22(01):433. 37978376 10.1186/s12912-023-01592-3 PMC10655287

[39] Wang XJ, Si LJ, Shi HM, et al. Survey of core competence of 546 nurses in a tertiary general hospital and analysis of their influencing factors[J]. Heilongjiang Med. 2024;48(01):65–7.

[40] Wang R, Chen S, Cong S, et al. Status and influencing factors of nursing and midwifery professionals’ core competence- a cross sectional study. J Nurs Manag. 2022;30(08):3891–9. 35213935 10.1111/jonm.13566

[41] Chen Q, Qiu DS, Zhang YW, et al. Analysis of the status quo and influencing factors of core competence of geriatric nursing for nurses in nursing institutions in Weifang City[J]. J Nurs. 2015;22(20):1–5.

[42] Chen HX, Yang X, Hu XY, et al. A survey and analysis on current status of the training and work for gerontological nurse specialists in 30 provinces in China. Chin J Nurs. 2021;56(09):1363–8.

[43] Yang MF, Zhou FH, Chen XY. Investigation and analysis of core competencies of nurses in geriatric care wards in Shanghai[J]. Electron J Practical Clin Nurs. 2017;2(51):156–7+159.

[44] Zamanzadeh V, Roshangar F, Fathi-Azar E, et al. Experiences of newly graduated nurses on strategies of gaining self-confidence during their initial work: a qualitative study. J Nurs Res. 2014;22(04):283–91. 25265368 10.1097/jnr.0000000000000050

[45] Spinelli C, Wisener M, Khoury B. Mindfulness training for healthcare professionals and trainees: a meta-analysis of randomized controlled trials. J Psychosom Res. 2019;120:29–38. 30929705 10.1016/j.jpsychores.2019.03.003

[46] Wang Q, Wang F, Zhang S, et al. Effects of a mindfulness-based interventions on stress, burnout in nurses: a systematic review and meta-analysis. Front Psychiatry. 2023;14:1218340. 37599884 10.3389/fpsyt.2023.1218340 PMC10434780

[47] Chen XZ, Liu QG, Meng FJ, et al. Correlation of nurses’ occupational coping self-efficacy with occupational stressors and core competencies[J]. Mod Clin Nurs. 2015;14(04):12–5.

[48] Wang J, Chen J, Zheng L, et al. Influence of psychological capital on core competency for new nurses. Plos One. 2023;18(08):e0289105.l 37561799 10.1371/journal.pone.0289105 PMC10414633

[49] Pisanti R, van der Doef M, et al. Occupational coping self-efficacy explains distress and well-being in nurses beyond psychosocial job characteristics. Front Psychol. 2015;6:1143. 26300827 10.3389/fpsyg.2015.01143 PMC4526791

[50] Tong Y, Wang T, Tong S, et al. Relationship among core competency, self-efficacy and transition shock in Chinese newly graduated nurses: a cross-sectional study. BMJ Open. 2024;14(04):e082865. 38569675 10.1136/bmjopen-2023-082865 PMC11146377

[51] Li X, Zhou M, Wang H, Hao W. Factors associated with core competencies of emergency-room nurses in tertiary hospitals in China. Jpn J Nurs Sci. 2020;17(03):e12337. 32239754 10.1111/jjns.12337

[52] Wang Y, Yang Y, Wang X, et al. Status and influencing factors of undergraduate midwifery students’ core competencies: a cross sectional study. Nurse Educ Today. 2024;133:106042. 37984053 10.1016/j.nedt.2023.106042

[53] Shahrour G, Dardas LA. Acute stress disorder, coping self-efficacy and subsequent psychological distress among nurses amid COVID-19. J Nurs Manag. 2020;28(07):1686–95. 32767827 10.1111/jonm.13124 PMC7436502

[54] Zhang WY, Mao YQ. How headmasters’ transformational leadership affects teachers’ organisational commitment-an empirical analysis based on mediating and moderating effects[J]. Educational Res. 2022;43(06):134–47.

[55] Zhang K, Pang Y. Progress and prospect of research on the mediating role mechanism of leadership style and employee initiative behaviour[J]. Contemp Econ Policy. 2024,46(07):77–88.

